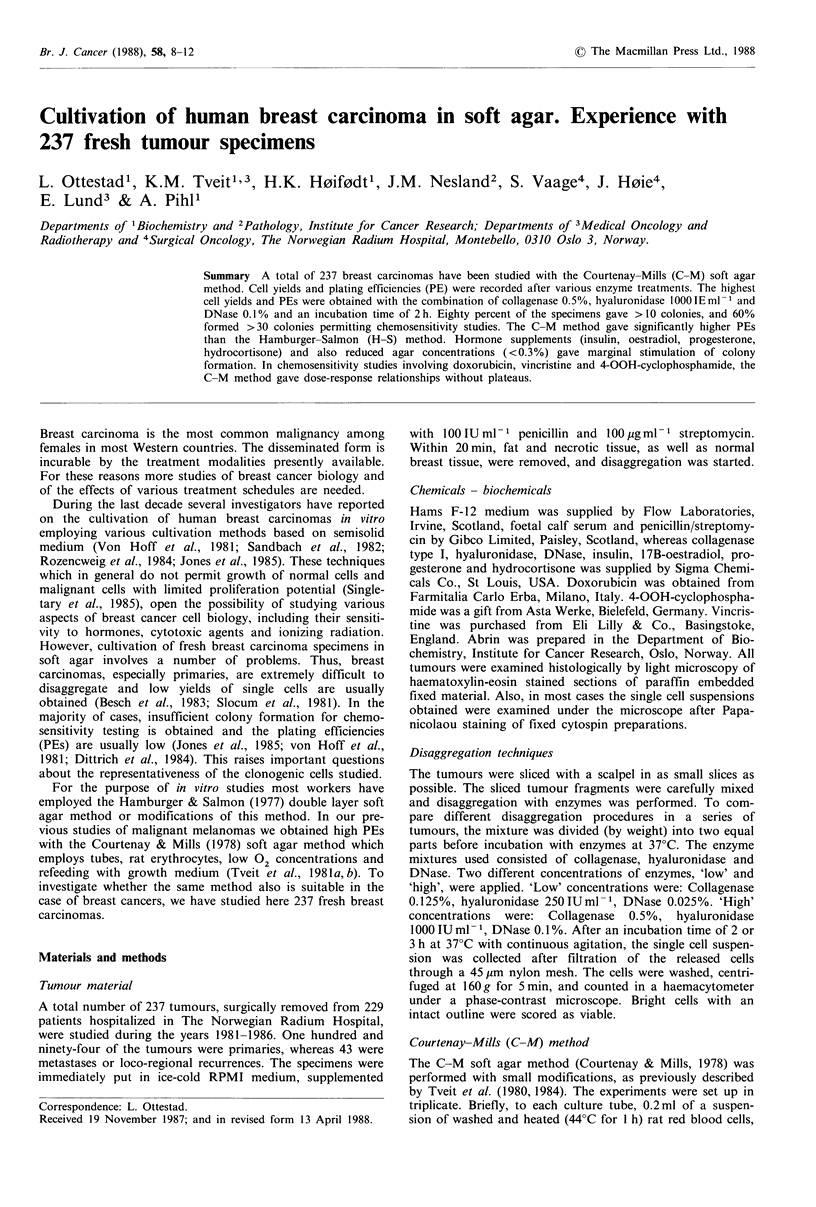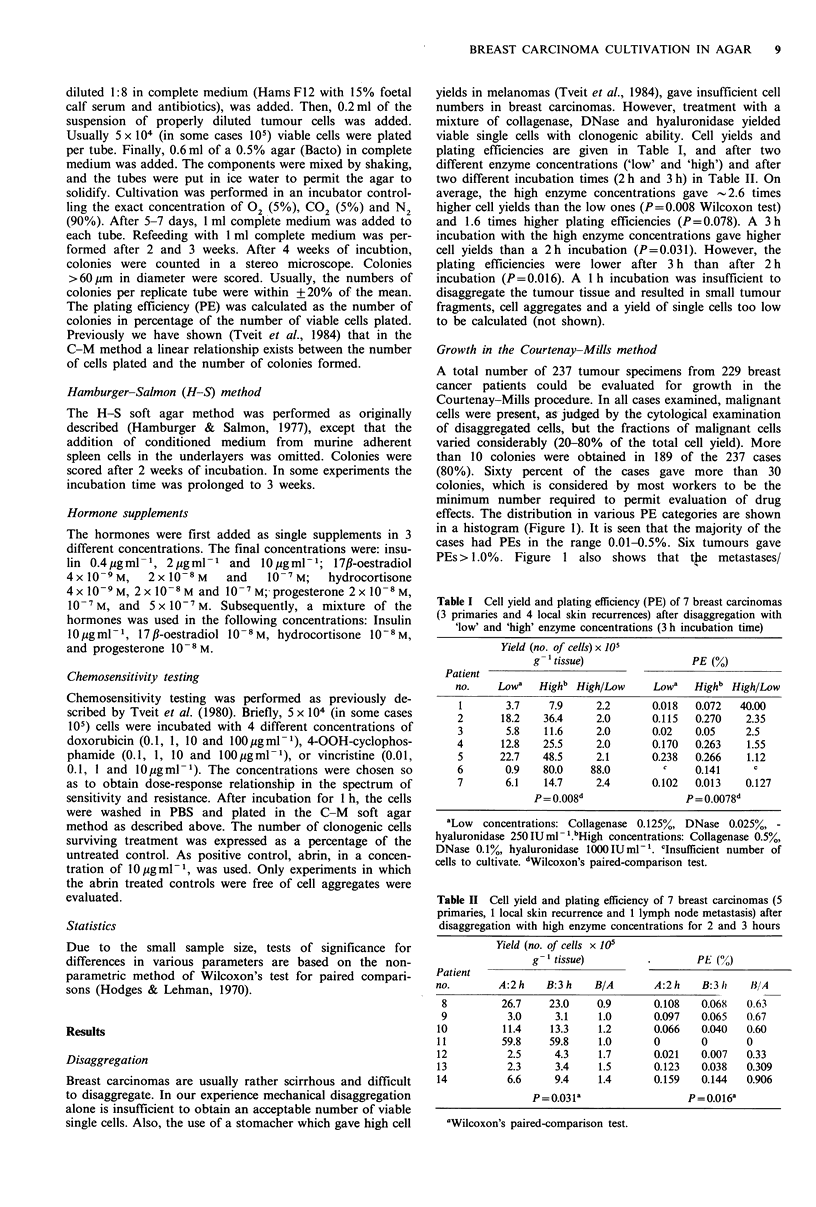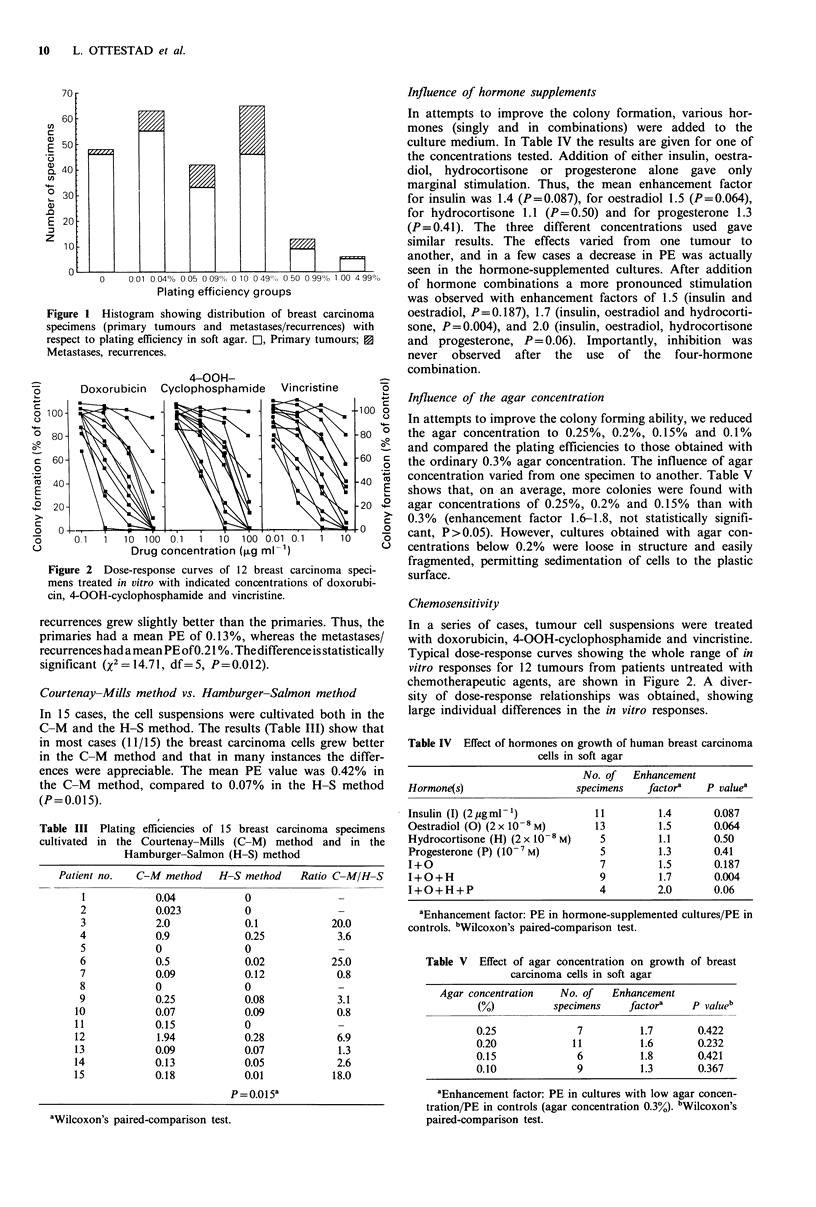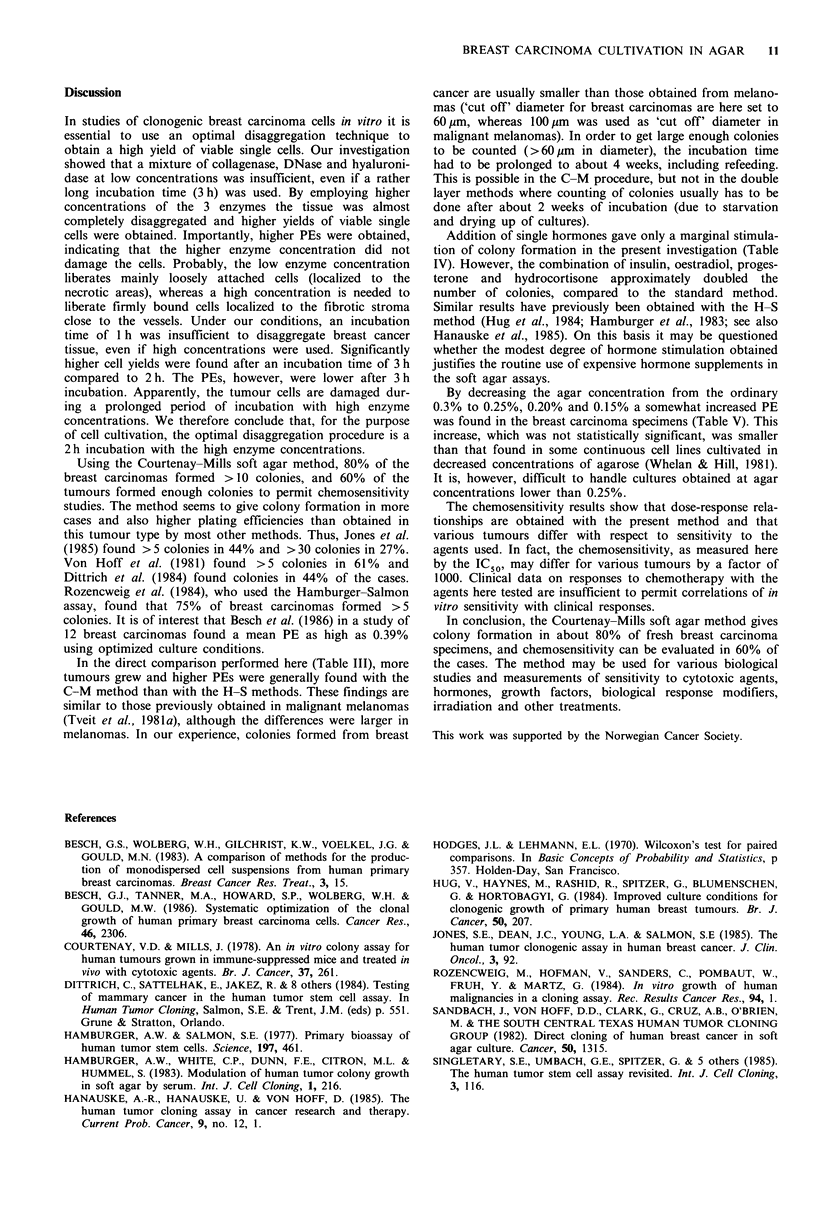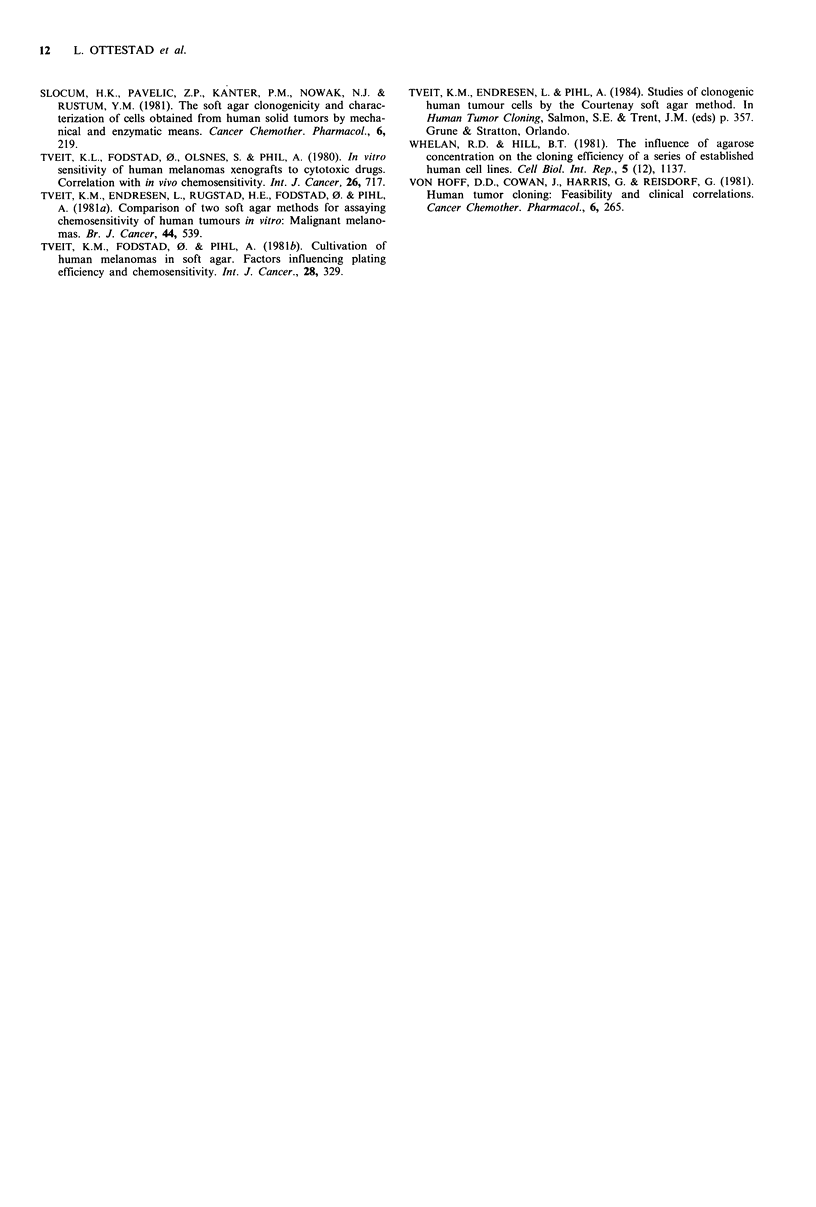# Cultivation of human breast carcinoma in soft agar. Experience with 237 fresh tumour specimens.

**DOI:** 10.1038/bjc.1988.151

**Published:** 1988-07

**Authors:** L. Ottestad, K. M. Tveit, H. K. HÃ¸ifÃ¸dt, J. M. Nesland, S. Vaage, J. HÃ¸ie, E. Lund, A. Pihl

**Affiliations:** Department of Biochemistry, Institute for Cancer Research, Oslo, Norway.

## Abstract

A total of 237 breast carcinomas have been studied with the Courtenay-Mills (C-M) soft agar method. Cell yields and plating efficiencies (PE) were recorded after various enzyme treatments. The highest cell yields and PEs were obtained with the combination of collagenase 0.5%, hyaluronidase 1000 IE ml-1 and DNase 0.1% and an incubation time of 2 h. Eighty percent of the specimens gave greater than 10 colonies, and 60% formed greater than 30 colonies permitting chemosensitivity studies. The C-M method gave significantly higher PEs than the Hamburger-Salmon (H-S) method. Hormone supplements (insulin, oestradiol, progesterone, hydrocortisone) and also reduced agar concentrations (less than 0.3%) gave marginal stimulation of colony formation. In chemosensitivity studies involving doxorubicin, vincristine and 4-OOH-cyclophosphamide, the C-M method gave dose-response relationships without plateaus.


*Br. J. Cancer* (1988), **58**, 8–12 © The Macmillan Press Ltd., 1988

### Cultivation of human breast carcinoma in soft agar. Experience with 237 fresh tumour specimens

L. Ottestad[1], K.M. Tveit[1,3], H.K. Høifødt[1], J.M. Nesland[2], S. Vaage[4], J. Høie[4], E. Lund[3] & A. Pihl[1]

*Departments of [1]Biochemistry and [2]Pathology, Institute for Cancer Research; Departments of [3]Medical Oncology and Radiotherapy and [4]Surgical Oncology, The Norwegian Radium Hospital, Montebello, 0310 Oslo 3, Norway.*

**Summary** A total of 237 breast carcinomas have been studied with the Courtenay–Mills (C–M) soft agar method. Cell yields and plating efficiencies (PE) were recorded after various enzyme treatments. The highest cell yields and PEs were obtained with the combination of collagenase 0.5%, hyaluronidase $1000\,IE\,ml^{-1}$ and DNase 0.1% and an incubation time of 2 h. Eighty percent of the specimens gave >10 colonies, and 60% formed >30 colonies permitting chemosensitivity studies. The C–M method gave significantly higher PEs than the Hamburger–Salmon (H–S) method. Hormone supplements (insulin, oestradiol, progesterone, hydrocortisone) and also reduced agar concentrations (<0.3%) gave marginal stimulation of colony formation. In chemosensitivity studies involving doxorubicin, vincristine and 4-OOH-cyclophosphamide, the C–M method gave dose-response relationships without plateaus.

Breast carcinoma is the most common malignancy among females in most Western countries. The disseminated form is incurable by the treatment modalities presently available. For these reasons more studies of breast cancer biology and of the effects of various treatment schedules are needed.

During the last decade several investigators have reported on the cultivation of human breast carcinomas *in vitro* employing various cultivation methods based on semisolid medium (Von Hoff *et al.*, 1981; Sandbach *et al.*, 1982; Rozencweig *et al.*, 1984; Jones *et al.*, 1985). These techniques which in general do not permit growth of normal cells and malignant cells with limited proliferation potential (Singletary *et al.*, 1985), open the possibility of studying various aspects of breast cancer cell biology, including their sensitivity to hormones, cytotoxic agents and ionizing radiation. However, cultivation of fresh breast carcinoma specimens in soft agar involves a number of problems. Thus, breast carcinomas, especially primaries, are extremely difficult to disaggregate and low yields of single cells are usually obtained (Besch *et al.*, 1983; Slocum *et al.*, 1981). In the majority of cases, insufficient colony formation for chemosensitivity testing is obtained and the plating efficiencies (PEs) are usually low (Jones *et al.*, 1985; von Hoff *et al.*, 1981; Dittrich *et al.*, 1984). This raises important questions about the representativeness of the clonogenic cells studied.

For the purpose of *in vitro* studies most workers have employed the Hamburger & Salmon (1977) double layer soft agar method or modifications of this method. In our previous studies of malignant melanomas we obtained high PEs with the Courtenay & Mills (1978) soft agar method which employs tubes, rat erythrocytes, low $O_2$ concentrations and refeeding with growth medium (Tveit *et al.*, 1981*a*, *b*). To investigate whether the same method also is suitable in the case of breast cancers, we have studied here 237 fresh breast carcinomas.

### Materials and methods

### *Tumour material*

A total number of 237 tumours, surgically removed from 229 patients hospitalized in The Norwegian Radium Hospital, were studied during the years 1981–1986. One hundred and ninety-four of the tumours were primaries, whereas 43 were metastases or loco-regional recurrences. The specimens were immediately put in ice-cold RPMI medium, supplemented with $100\,IU\,ml^{-1}$ penicillin and $100\,\mu g\,ml^{-1}$ streptomycin. Within 20 min, fat and necrotic tissue, as well as normal breast tissue, were removed, and disaggregation was started.

### *Chemicals – biochemicals*

Hams F-12 medium was supplied by Flow Laboratories, Irvine, Scotland, foetal calf serum and penicillin/streptomycin by Gibco Limited, Paisley, Scotland, whereas collagenase type I, hyaluronidase, DNase, insulin, 17B-oestradiol, progesterone and hydrocortisone was supplied by Sigma Chemicals Co., St Louis, USA. Doxorubicin was obtained from Farmitalia Carlo Erba, Milano, Italy. 4-OOH-cyclophosphamide was a gift from Asta Werke, Bielefeld, Germany. Vincristine was purchased from Eli Lilly & Co., Basingstoke, England. Abrin was prepared in the Department of Biochemistry, Institute for Cancer Research, Oslo, Norway. All tumours were examined histologically by light microscopy of haematoxylin-eosin stained sections of paraffin embedded fixed material. Also, in most cases the single cell suspensions obtained were examined under the microscope after Papanicolaou staining of fixed cytospin preparations.

### *Disaggregation techniques*

The tumours were sliced with a scalpel in as small slices as possible. The sliced tumour fragments were carefully mixed and disaggregation with enzymes was performed. To compare different disaggregation procedures in a series of tumours, the mixture was divided (by weight) into two equal parts before incubation with enzymes at 37°C. The enzyme mixtures used consisted of collagenase, hyaluronidase and DNase. Two different concentrations of enzymes, 'low' and 'high', were applied. 'Low' concentrations were: Collagenase 0.125%, hyaluronidase $250\,IU\,ml^{-1}$, DNase 0.025%. 'High' concentrations were: Collagenase 0.5%, hyaluronidase $1000\,IU\,ml^{-1}$, DNase 0.1%. After an incubation time of 2 or 3 h at 37°C with continuous agitation, the single cell suspension was collected after filtration of the released cells through a $45\,\mu m$ nylon mesh. The cells were washed, centrifuged at $160\,g$ for 5 min, and counted in a haemacytometer under a phase-contrast microscope. Bright cells with an intact outline were scored as viable.

### *Courtenay–Mills (C–M) method*

The C–M soft agar method (Courtenay & Mills, 1978) was performed with small modifications, as previously described by Tveit *et al.* (1980, 1984). The experiments were set up in triplicate. Briefly, to each culture tube, 0.2 ml of a suspension of washed and heated (44°C for 1 h) rat red blood cells,

Correspondence: L. Ottestad.
Received 19 November 1987; and in revised form 13 April 1988.

diluted 1:8 in complete medium (Hams F12 with 15% foetal calf serum and antibiotics), was added. Then, 0.2 ml of the suspension of properly diluted tumour cells was added. Usually $5 \times 10^4$ (in some cases $10^5$) viable cells were plated per tube. Finally, 0.6 ml of a 0.5% agar (Bacto) in complete medium was added. The components were mixed by shaking, and the tubes were put in ice water to permit the agar to solidify. Cultivation was performed in an incubator controlling the exact concentration of $O_2$ (5%), $CO_2$ (5%) and $N_2$ (90%). After 5–7 days, 1 ml complete medium was added to each tube. Refeeding with 1 ml complete medium was performed after 2 and 3 weeks. After 4 weeks of incubtion, colonies were counted in a stereo microscope. Colonies $>60\,\mu m$ in diameter were scored. Usually, the numbers of colonies per replicate tube were within $\pm 20\%$ of the mean. The plating efficiency (PE) was calculated as the number of colonies in percentage of the number of viable cells plated. Previously we have shown (Tveit *et al.*, 1984) that in the C–M method a linear relationship exists between the number of cells plated and the number of colonies formed.

### *Hamburger–Salmon (H–S) method*

The H-S soft agar method was performed as originally described (Hamburger & Salmon, 1977), except that the addition of conditioned medium from murine adherent spleen cells in the underlayers was omitted. Colonies were scored after 2 weeks of incubation. In some experiments the incubation time was prolonged to 3 weeks.

### *Hormone supplements*

The hormones were first added as single supplements in 3 different concentrations. The final concentrations were: insulin $0.4\,\mu g\,ml^{-1}$, $2\,\mu g\,ml^{-1}$ and $10\,\mu g\,ml^{-1}$; 17β-oestradiol $4 \times 10^{-9}$ M, $2 \times 10^{-8}$ M and $10^{-7}$ M; hydrocortisone $4 \times 10^{-9}$ M, $2 \times 10^{-8}$ M and $10^{-7}$ M; progesterone $2 \times 10^{-8}$ M, $10^{-7}$ M, and $5 \times 10^{-7}$ M. Subsequently, a mixture of the hormones was used in the following concentrations: Insulin $10\,\mu g\,ml^{-1}$, 17β-oestradiol $10^{-8}$ M, hydrocortisone $10^{-8}$ M, and progesterone $10^{-8}$ M.

### *Chemosensitivity testing*

Chemosensitivity testing was performed as previously described by Tveit *et al.* (1980). Briefly, $5 \times 10^4$ (in some cases $10^5$) cells were incubated with 4 different concentrations of doxorubicin (0.1, 1, 10 and $100\,\mu g\,ml^{-1}$), 4-OOH-cyclophosphamide (0.1, 1, 10 and $100\,\mu g\,ml^{-1}$), or vincristine (0.01, 0.1, 1 and $10\,\mu g\,ml^{-1}$). The concentrations were chosen so as to obtain dose-response relationship in the spectrum of sensitivity and resistance. After incubation for 1 h, the cells were washed in PBS and plated in the C–M soft agar method as described above. The number of clonogenic cells surviving treatment was expressed as a percentage of the untreated control. As positive control, abrin, in a concentration of $10\,\mu g\,ml^{-1}$, was used. Only experiments in which the abrin treated controls were free of cell aggregates were evaluated.

### *Statistics*

Due to the small sample size, tests of significance for differences in various parameters are based on the non-parametric method of Wilcoxon's test for paired comparisons (Hodges & Lehman, 1970).

### Results

### *Disaggregation*

Breast carcinomas are usually rather scirrhous and difficult to disaggregate. In our experience mechanical disaggregation alone is insufficient to obtain an acceptable number of viable single cells. Also, the use of a stomacher which gave high cell yields in melanomas (Tveit *et al.*, 1984), gave insufficient cell numbers in breast carcinomas. However, treatment with a mixture of collagenase, DNase and hyaluronidase yielded viable single cells with clonogenic ability. Cell yields and plating efficiencies are given in Table I, and after two different enzyme concentrations ('low' and 'high') and after two different incubation times (2 h and 3 h) in Table II. On average, the high enzyme concentrations gave ~2.6 times higher cell yields than the low ones ($P=0.008$ Wilcoxon test) and 1.6 times higher plating efficiencies ($P=0.078$). A 3 h incubation with the high enzyme concentrations gave higher cell yields than a 2 h incubation ($P=0.031$). However, the plating efficiencies were lower after 3 h than after 2 h incubation ($P=0.016$). A 1 h incubation was insufficient to disaggregate the tumour tissue and resulted in small tumour fragments, cell aggregates and a yield of single cells too low to be calculated (not shown).

### *Growth in the Courtenay–Mills method*

A total number of 237 tumour specimens from 229 breast cancer patients could be evaluated for growth in the Courtenay–Mills procedure. In all cases examined, malignant cells were present, as judged by the cytological examination of disaggregated cells, but the fractions of malignant cells varied considerably (20–80% of the total cell yield). More than 10 colonies were obtained in 189 of the 237 cases (80%). Sixty percent of the cases gave more than 30 colonies, which is considered by most workers to be the minimum number required to permit evaluation of drug effects. The distribution in various PE categories are shown in a histogram (Figure 1). It is seen that the majority of the cases had PEs in the range 0.01–0.5%. Six tumours gave PEs > 1.0%. Figure 1 also shows that the metastases/

**Table I** Cell yield and plating efficiency (PE) of 7 breast carcinomas (3 primaries and 4 local skin recurrences) after disaggregation with 'low' and 'high' enzyme concentrations (3 h incubation time)

| Patient no. | *Yield (no. of cells)* $\times 10^5$ $g^{-1}$ *tissue)* Low[a] | High[b] | High/Low | *PE (%)* Low[a] | High[b] | High/Low |
|---|---|---|---|---|---|---|
| 1 | 3.7 | 7.9 | 2.2 | 0.018 | 0.072 | 40.00 |
| 2 | 18.2 | 36.4 | 2.0 | 0.115 | 0.270 | 2.35 |
| 3 | 5.8 | 11.6 | 2.0 | 0.02 | 0.05 | 2.5 |
| 4 | 12.8 | 25.5 | 2.0 | 0.170 | 0.263 | 1.55 |
| 5 | 22.7 | 48.5 | 2.1 | 0.238 | 0.266 | 1.12 |
| 6 | 0.9 | 80.0 | 88.0 | [c] | 0.141 | [c] |
| 7 | 6.1 | 14.7 | 2.4 | 0.102 | 0.013 | 0.127 |
| | | $P=0.008$[d] | | | $P=0.0078$[d] | |

[a]Low concentrations: Collagenase 0.125%, DNase 0.025%, - hyaluronidase $250\,IU\,ml^{-1}$.[b]High concentrations: Collagenase 0.5%, DNase 0.1%, hyaluronidase $1000\,IU\,ml^{-1}$. [c]Insufficient number of cells to cultivate. [d]Wilcoxon's paired-comparison test.

**Table II** Cell yield and plating efficiency of 7 breast carcinomas (5 primaries, 1 local skin recurrence and 1 lymph node metastasis) after disaggregation with high enzyme concentrations for 2 and 3 hours

| Patient no. | *Yield (no. of cells* $\times 10^5$ $g^{-1}$ *tissue)* A:2 h | B:3 h | B/A | *PE (%)* A:2 h | B:3 h | B/A |
|---|---|---|---|---|---|---|
| 8 | 26.7 | 23.0 | 0.9 | 0.108 | 0.068 | 0.63 |
| 9 | 3.0 | 3.1 | 1.0 | 0.097 | 0.065 | 0.67 |
| 10 | 11.4 | 13.3 | 1.2 | 0.066 | 0.040 | 0.60 |
| 11 | 59.8 | 59.8 | 1.0 | 0 | 0 | 0 |
| 12 | 2.5 | 4.3 | 1.7 | 0.021 | 0.007 | 0.33 |
| 13 | 2.3 | 3.4 | 1.5 | 0.123 | 0.038 | 0.309 |
| 14 | 6.6 | 9.4 | 1.4 | 0.159 | 0.144 | 0.906 |
| | | $P=0.031$[a] | | | $P=0.016$[a] | |

[a]Wilcoxon's paired-comparison test.

**Figure 1** Histogram showing distribution of breast carcinoma specimens (primary tumours and metastases/recurrences) with respect to plating efficiency in soft agar. □, Primary tumours; ▨ Metastases, recurrences.

**Figure 2** Dose-response curves of 12 breast carcinoma specimens treated *in vitro* with indicated concentrations of doxorubicin, 4-OOH-cyclophosphamide and vincristine.

recurrences grew slightly better than the primaries. Thus, the primaries had a mean PE of 0.13%, whereas the metastases/recurrences had a mean PE of 0.21%. The difference is statistically significant ($\chi^2=14.71$, df=5, $P=0.012$).

*Courtenay–Mills method vs. Hamburger–Salmon method*

In 15 cases, the cell suspensions were cultivated both in the C–M and the H–S method. The results (Table III) show that in most cases (11/15) the breast carcinoma cells grew better in the C–M method and that in many instances the differences were appreciable. The mean PE value was 0.42% in the C–M method, compared to 0.07% in the H–S method ($P=0.015$).

**Table III** Plating efficiencies of 15 breast carcinoma specimens cultivated in the Courtenay–Mills (C–M) method and in the Hamburger–Salmon (H–S) method

| *Patient no.* | *C–M method* | *H–S method* | *Ratio C–M/H–S* |
|---|---|---|---|
| 1 | 0.04 | 0 | – |
| 2 | 0.023 | 0 | – |
| 3 | 2.0 | 0.1 | 20.0 |
| 4 | 0.9 | 0.25 | 3.6 |
| 5 | 0 | 0 | – |
| 6 | 0.5 | 0.02 | 25.0 |
| 7 | 0.09 | 0.12 | 0.8 |
| 8 | 0 | 0 | – |
| 9 | 0.25 | 0.08 | 3.1 |
| 10 | 0.07 | 0.09 | 0.8 |
| 11 | 0.15 | 0 | – |
| 12 | 1.94 | 0.28 | 6.9 |
| 13 | 0.09 | 0.07 | 1.3 |
| 14 | 0.13 | 0.05 | 2.6 |
| 15 | 0.18 | 0.01 | 18.0 |
| | | $P=0.015$[a] | |

[a]Wilcoxon's paired-comparison test.

*Influence of hormone supplements*

In attempts to improve the colony formation, various hormones (singly and in combinations) were added to the culture medium. In Table IV the results are given for one of the concentrations tested. Addition of either insulin, oestradiol, hydrocortisone or progesterone alone gave only marginal stimulation. Thus, the mean enhancement factor for insulin was 1.4 ($P=0.087$), for oestradiol 1.5 ($P=0.064$), for hydrocortisone 1.1 ($P=0.50$) and for progesterone 1.3 ($P=0.41$). The three different concentrations used gave similar results. The effects varied from one tumour to another, and in a few cases a decrease in PE was actually seen in the hormone-supplemented cultures. After addition of hormone combinations a more pronounced stimulation was observed with enhancement factors of 1.5 (insulin and oestradiol, $P=0.187$), 1.7 (insulin, oestradiol and hydrocortisone, $P=0.004$), and 2.0 (insulin, oestradiol, hydrocortisone and progesterone, $P=0.06$). Importantly, inhibition was never observed after the use of the four-hormone combination.

*Influence of the agar concentration*

In attempts to improve the colony forming ability, we reduced the agar concentration to 0.25%, 0.2%, 0.15% and 0.1% and compared the plating efficiencies to those obtained with the ordinary 0.3% agar concentration. The influence of agar concentration varied from one specimen to another. Table V shows that, on an average, more colonies were found with agar concentrations of 0.25%, 0.2% and 0.15% than with 0.3% (enhancement factor 1.6–1.8, not statistically significant, $P>0.05$). However, cultures obtained with agar concentrations below 0.2% were loose in structure and easily fragmented, permitting sedimentation of cells to the plastic surface.

*Chemosensitivity*

In a series of cases, tumour cell suspensions were treated with doxorubicin, 4-OOH-cyclophosphamide and vincristine. Typical dose-response curves showing the whole range of *in vitro* responses for 12 tumours from patients untreated with chemotherapeutic agents, are shown in Figure 2. A diversity of dose-response relationships was obtained, showing large individual differences in the *in vitro* responses.

**Table IV** Effect of hormones on growth of human breast carcinoma cells in soft agar

| *Hormone(s)* | *No. of specimens* | *Enhancement factor*[a] | *P value*[a] |
|---|---|---|---|
| Insulin (I) (2 μg ml$^{-1}$) | 11 | 1.4 | 0.087 |
| Oestradiol (O) ($2\times10^{-8}$ M) | 13 | 1.5 | 0.064 |
| Hydrocortisone (H) ($2\times10^{-8}$ M) | 5 | 1.1 | 0.50 |
| Progesterone (P) ($10^{-7}$ M) | 5 | 1.3 | 0.41 |
| I+O | 7 | 1.5 | 0.187 |
| I+O+H | 9 | 1.7 | 0.004 |
| I+O+H+P | 4 | 2.0 | 0.06 |

[a]Enhancement factor: PE in hormone-supplemented cultures/PE in controls. [b]Wilcoxon's paired-comparison test.

**Table V** Effect of agar concentration on growth of breast carcinoma cells in soft agar

| *Agar concentration (%)* | *No. of specimens* | *Enhancement factor*[a] | *P value*[b] |
|---|---|---|---|
| 0.25 | 7 | 1.7 | 0.422 |
| 0.20 | 11 | 1.6 | 0.232 |
| 0.15 | 6 | 1.8 | 0.421 |
| 0.10 | 9 | 1.3 | 0.367 |

[a]Enhancement factor: PE in cultures with low agar concentration/PE in controls (agar concentration 0.3%). [b]Wilcoxon's paired-comparison test.

### Discussion

In studies of clonogenic breast carcinoma cells *in vitro* it is essential to use an optimal disaggregation technique to obtain a high yield of viable single cells. Our investigation showed that a mixture of collagenase, DNase and hyaluronidase at low concentrations was insufficient, even if a rather long incubation time (3 h) was used. By employing higher concentrations of the 3 enzymes the tissue was almost completely disaggregated and higher yields of viable single cells were obtained. Importantly, higher PEs were obtained, indicating that the higher enzyme concentration did not damage the cells. Probably, the low enzyme concentration liberates mainly loosely attached cells (localized to the necrotic areas), whereas a high concentration is needed to liberate firmly bound cells localized to the fibrotic stroma close to the vessels. Under our conditions, an incubation time of 1 h was insufficient to disaggregate breast cancer tissue, even if high concentrations were used. Significantly higher cell yields were found after an incubation time of 3 h compared to 2 h. The PEs, however, were lower after 3 h incubation. Apparently, the tumour cells are damaged during a prolonged period of incubation with high enzyme concentrations. We therefore conclude that, for the purpose of cell cultivation, the optimal disaggregation procedure is a 2 h incubation with the high enzyme concentrations.

Using the Courtenay–Mills soft agar method, 80% of the breast carcinomas formed >10 colonies, and 60% of the tumours formed enough colonies to permit chemosensitivity studies. The method seems to give colony formation in more cases and also higher plating efficiencies than obtained in this tumour type by most other methods. Thus, Jones *et al.* (1985) found >5 colonies in 44% and >30 colonies in 27%. Von Hoff *et al.* (1981) found >5 colonies in 61% and Dittrich *et al.* (1984) found colonies in 44% of the cases. Rozencweig *et al.* (1984), who used the Hamburger–Salmon assay, found that 75% of breast carcinomas formed >5 colonies. It is of interest that Besch *et al.* (1986) in a study of 12 breast carcinomas found a mean PE as high as 0.39% using optimized culture conditions.

In the direct comparison performed here (Table III), more tumours grew and higher PEs were generally found with the C–M method than with the H–S methods. These findings are similar to those previously obtained in malignant melanomas (Tveit *et al.*, 1981*a*), although the differences were larger in melanomas. In our experience, colonies formed from breast cancer are usually smaller than those obtained from melanomas ('cut off' diameter for breast carcinomas are here set to 60 $\mu$m, whereas 100 $\mu$m was used as 'cut off' diameter in malignant melanomas). In order to get large enough colonies to be counted (>60 $\mu$m in diameter), the incubation time had to be prolonged to about 4 weeks, including refeeding. This is possible in the C–M procedure, but not in the double layer methods where counting of colonies usually has to be done after about 2 weeks of incubation (due to starvation and drying up of cultures).

Addition of single hormones gave only a marginal stimulation of colony formation in the present investigation (Table IV). However, the combination of insulin, oestradiol, progesterone and hydrocortisone approximately doubled the number of colonies, compared to the standard method. Similar results have previously been obtained with the H–S method (Hug *et al.*, 1984; Hamburger *et al.*, 1983; see also Hanauske *et al.*, 1985). On this basis it may be questioned whether the modest degree of hormone stimulation obtained justifies the routine use of expensive hormone supplements in the soft agar assays.

By decreasing the agar concentration from the ordinary 0.3% to 0.25%, 0.20% and 0.15% a somewhat increased PE was found in the breast carcinoma specimens (Table V). This increase, which was not statistically significant, was smaller than that found in some continuous cell lines cultivated in decreased concentrations of agarose (Whelan & Hill, 1981). It is, however, difficult to handle cultures obtained at agar concentrations lower than 0.25%.

The chemosensitivity results show that dose-response relationships are obtained with the present method and that various tumours differ with respect to sensitivity to the agents used. In fact, the chemosensitivity, as measured here by the $IC_{50}$, may differ for various tumours by a factor of 1000. Clinical data on responses to chemotherapy with the agents here tested are insufficient to permit correlations of *in vitro* sensitivity with clinical responses.

In conclusion, the Courtenay–Mills soft agar method gives colony formation in about 80% of fresh breast carcinoma specimens, and chemosensitivity can be evaluated in 60% of the cases. The method may be used for various biological studies and measurements of sensitivity to cytotoxic agents, hormones, growth factors, biological response modifiers, irradiation and other treatments.

This work was supported by the Norwegian Cancer Society.

### References

BESCH, G.S., WOLBERG, W.H., GILCHRIST, K.W., VOELKEL, J.G. & GOULD, M.N. (1983). A comparison of methods for the production of monodispersed cell suspensions from human primary breast carcinomas. *Breast Cancer Res. Treat.*, **3**, 15.

BESCH, G.J., TANNER, M.A., HOWARD, S.P., WOLBERG, W.H. & GOULD, M.W. (1986). Systematic optimization of the clonal growth of human primary breast carcinoma cells. *Cancer Res.*, **46**, 2306.

COURTENAY, V.D. & MILLS, J. (1978). An *in vitro* colony assay for human tumours grown in immune-suppressed mice and treated *in vivo* with cytotoxic agents. *Br. J. Cancer*, **37**, 261.

DITTRICH, C., SATTELHAK, E., JAKEZ, R. & 8 others (1984). Testing of mammary cancer in the human tumor stem cell assay. In *Human Tumor Cloning*, Salmon, S.E. & Trent, J.M. (eds) p. 551. Grune & Stratton, Orlando.

HAMBURGER, A.W. & SALMON, S.E. (1977). Primary bioassay of human tumor stem cells. *Science*, **197**, 461.

HAMBURGER, A.W., WHITE, C.P., DUNN, F.E., CITRON, M.L. & HUMMEL, S. (1983). Modulation of human tumor colony growth in soft agar by serum. *Int. J. Cell Cloning*, **1**, 216.

HANAUSKE, A.-R., HANAUSKE, U. & VON HOFF, D. (1985). The human tumor cloning assay in cancer research and therapy. *Current Prob. Cancer*, **9**, no. 12, 1.

HODGES, J.L. & LEHMANN, E.L. (1970). Wilcoxon's test for paired comparisons. In *Basic Concepts of Probability and Statistics*, p 357. Holden-Day, San Francisco.

HUG, V., HAYNES, M., RASHID, R., SPITZER, G., BLUMENSCHEN, G. & HORTOBAGYI, G. (1984). Improved culture conditions for clonogenic growth of primary human breast tumours. *Br. J. Cancer*, **50**, 207.

JONES, S.E., DEAN, J.C., YOUNG, L.A. & SALMON, S.E (1985). The human tumor clonogenic assay in human breast cancer. *J. Clin. Oncol.*, **3**, 92.

ROZENCWEIG, M., HOFMAN, V., SANDERS, C., POMBAUT, W., FRUH, Y. & MARTZ, G. (1984). *In vitro* growth of human malignancies in a cloning assay. *Rec. Results Cancer Res.*, **94**, 1.

SANDBACH, J., VON HOFF, D.D., CLARK, G., CRUZ, A.B., O'BRIEN, M. & THE SOUTH CENTRAL TEXAS HUMAN TUMOR CLONING GROUP (1982). Direct cloning of human breast cancer in soft agar culture. *Cancer*, **50**, 1315.

SINGLETARY, S.E., UMBACH, G.E., SPITZER, G. & 5 others (1985). The human tumor stem cell assay revisited. *Int. J. Cell Cloning*, **3**, 116.

SLOCUM, H.K., PAVELIC, Z.P., KANTER, P.M., NOWAK, N.J. & RUSTUM, Y.M. (1981). The soft agar clonogenicity and characterization of cells obtained from human solid tumors by mechanical and enzymatic means. *Cancer Chemother. Pharmacol.*, **6**, 219.

TVEIT, K.L., FODSTAD, Ø., OLSNES, S. & PHIL, A. (1980). *In vitro* sensitivity of human melanomas xenografts to cytotoxic drugs. Correlation with *in vivo* chemosensitivity. *Int. J. Cancer,* **26,** 717.

TVEIT, K.M., ENDRESEN, L., RUGSTAD, H.E., FODSTAD, Ø. & PIHL, A. (1981*a*). Comparison of two soft agar methods for assaying chemosensitivity of human tumours *in vitro*: Malignant melanomas. *Br. J. Cancer*, **44,** 539.

TVEIT, K.M., FODSTAD, Ø. & PIHL, A. (1981*b*). Cultivation of human melanomas in soft agar. Factors influencing plating efficiency and chemosensitivity. *Int. J. Cancer.*, **28,** 329.

TVEIT, K.M., ENDRESEN, L. & PIHL, A. (1984). Studies of clonogenic human tumour cells by the Courtenay soft agar method. In *Human Tumor Cloning*, Salmon, S.E. & Trent, J.M. (eds) p. 357. Grune & Stratton, Orlando.

WHELAN, R.D. & HILL, B.T. (1981). The influence of agarose concentration on the cloning efficiency of a series of established human cell lines. *Cell Biol. Int. Rep.*, **5** (12), 1137.

VON HOFF, D.D., COWAN, J., HARRIS, G. & REISDORF, G. (1981). Human tumor cloning: Feasibility and clinical correlations. *Cancer Chemother. Pharmacol.*, **6,** 265.